# Genomic diversity of *Salmonella enterica -*The UoWUCC 10K genomes project

**DOI:** 10.12688/wellcomeopenres.16291.2

**Published:** 2021-02-01

**Authors:** Mark Achtman, Zhemin Zhou, Nabil-Fareed Alikhan, William Tyne, Julian Parkhill, Martin Cormican, Chien-Shun Chiou, Mia Torpdahl, Eva Litrup, Deirdre M. Prendergast, John E. Moore, Sam Strain, Christian Kornschober, Richard Meinersmann, Alexandra Uesbeck, François-Xavier Weill, Aidan Coffey, Helene Andrews-Polymenis, Roy Curtiss 3rd, Séamus Fanning

**Affiliations:** 1 Warwick Medical School, University of Warwick, Coventry, CV4 7AL, UK; 2 Department of Veterinary Medicine, University of Cambridge, Cambridge, CB3 0ES, UK; 3 National Salmonella, Shigella and Listeria Reference Laboratory, Galway, H91 YR71, Ireland; 4 Central Regional Laboratory, Center for Diagnostics and Vaccine Development, Centers for Disease Control, Taichung, None, Taiwan; 5 Statens Serum Institut, Copenhagen S, DK-2300, Denmark; 6 Backweston complex, Department of Agriculture, Food and the Marine (DAFM), Celbridge, Co. Kildare, W23 X3PH, Ireland; 7 Northern Ireland Public Health Laboratory, Department of Bacteriology, Belfast City Hospital, Belfast, BT9 7AD, UK; 8 Animal Health and Welfare NI, Dungannon, BT71 6JT, UK; 9 Institute for Medical Microbiology and Hygiene, Austrian Agency for Health and Food Safety (AGES), Graz, 8010, Austria; 10 US National Poultry Research Center, USDA Agricultural Research Service, Athens, GA, 30605, USA; 11 Institute for Medical Microbiology, Immunology, and Hygiene, University of Cologne, Cologne, 50935, Germany; 12 Unité des bactéries pathogènes entériques, Institut Pasteur, Paris, cedex 15, France; 13 Cork Institute of Technology, Cork, T12P928, Ireland; 14 Dept. of Microbial Pathogenesis and Immunology, College of Medicine Texas A&M University, Bryan, TX, 77807, USA; 15 Dept. of Infectious Diseases & Immunology, College of Veterinary Medicine, University of Florida, Gainesville, Florida, 32611, USA; 16 UCD-Centre for Food Safety, University College Dublin, Dublin, D04 N2E5, Ireland

**Keywords:** Salmonella, Large scale genomic database, High throughput sequencing, Population genomics

## Abstract

**Background: **Most publicly available genomes of *Salmonella enterica* are from human disease in the US and the UK, or from domesticated animals in the US.

**Methods: **Here we describe a historical collection of 10,000 strains isolated between 1891-2010 in 73 different countries. They encompass a broad range of sources, ranging from rivers through reptiles to the diversity of all *S. enterica *isolated on the island of Ireland between 2000 and 2005. Genomic DNA was isolated, and sequenced by Illumina short read sequencing.

**Results:** The short reads are publicly available in the Short Reads Archive. They were also uploaded to EnteroBase, which assembled and annotated draft genomes. 9769 draft genomes which passed quality control were genotyped with multiple levels of multilocus sequence typing, and used to predict serovars. Genomes were assigned to hierarchical clusters on the basis of numbers of pair-wise allelic differences in core genes, which were mapped to genetic Lineages within phylogenetic trees.

**Conclusions:** The University of Warwick/University College Cork (UoWUCC) project greatly extends the geographic sources, dates and core genomic diversity of publicly available *S. enterica *genomes. We illustrate these features by an overview of core genomic Lineages within 33,000 publicly available *Salmonella *genomes whose strains were isolated before 2011. We also present detailed examinations of HC400, HC900 and HC2000 hierarchical clusters within exemplar Lineages, including serovars Typhimurium, Enteritidis and Mbandaka. These analyses confirm the polyphyletic nature of multiple serovars while showing that discrete clusters with geographical specificity can be reliably recognized by hierarchical clustering approaches. The results also demonstrate that the genomes sequenced here provide an important counterbalance to the sampling bias which is so dominant in current genomic sequencing.

## Introduction

*Salmonella enterica* is the one of the four global causes of diarrhoeal diseases in humans ( World Health Organization Fact Sheets, 2018), and has been estimated to be responsible for 94 million annual cases of nontyphoidal gastroenteritis ( Majowicz *et al.*, 2010). Most cases of salmonellosis are mild but the infections can be life-threatening, especially when salmonellosis manifests as typhoid fever caused by serovar Typhi ( Wong *et al.*, 2016), enteric fever due to serovars Paratyphi A or Paratyphi C ( Zhou *et al.*, 2014; Zhou *et al.*, 2018b), or extra-intestinal disease with serovars Choleraesuis ( Zhou *et al.*, 2018b) or Typhimurium ( Kingsley *et al.*, 2009; GBD 2017 Non-Typhoidal Salmonella Invasive Disease Collaborators, 2019). *S. enterica* also infects domesticated animals in large numbers, and was the primary cause of food-borne outbreaks reported in Europe ( European Food Safety Authority, 2007), leading to European regulations intended to reduce the numbers of animal herds contaminated with *Salmonella* (Regulation (EC) No 2160/2003).

The volume of bacterial genome sequencing is increasing dramatically. Since 2012, unprecedentedly large numbers of *Salmonella* genomes were sequenced by the Sanger Institute ( Feasey *et al.*, 2016; Wong *et al.*, 2016), the Food and Drug Administration ( Feldgarden *et al.*, 2019), CDC/PulseNet International ( Gerner-Smidt *et al.*, 2019; Nadon *et al.*, 2017) and Public Health England ( Ashton *et al.*, 2016; Waldram *et al.*, 2018). In August 2020, EnteroBase ( Alikhan *et al.*, 2018; Zhou *et al.*, 2020a) contained >260,000 *Salmonella* genomes which had been assembled from sequence reads in the public short read archives, or uploaded by its users. However, the global population genetic diversity of *Salmonella* encompassed by these genomes is not necessarily representative of total global diversity. Almost all of the bacterial strains were sequenced for epidemiological tracking of the sources of food-borne diseases. Most of them were from human infections in North America and England. Similarly, almost all public *Salmonella* genomes from domesticated animals are from North America, which causes even greater sample bias.

Serovars Typhi, Paratyphi A and Paratyphi C are specific for humans, and other serovars show signs of adaptation to other hosts ( Baumler *et al.*, 1998; Kingsley & Baumler, 2000). However, only limited data are available for most other serovars and from inter-continental comparisons ( Cheng *et al.*, 2019). We note that *S. enterica* can be isolated from rivers, ponds and drinking water ( Meinersmann *et al.*, 2008; Uesbeck, 2009; Walters *et al.*, 2011; Walters *et al.*, 2013) as well as salt water ( Mannas *et al.*, 2014; Martinez-Urtaza *et al.*, 2004). Reptiles are often infected by *Salmonella* ( Corrente *et al.*, 2017; Kanagarajah *et al.*, 2018; Mukherjee *et al.*, 2019; Pulford *et al.*, 2019), and *S. enterica* strains can invade plant cells, and survive in soil ( Dyda *et al.*, 2020; Jechalke *et al.*, 2019; Schikora *et al.*, 2012). The degree of overlap between bacterial populations from those sources and those that infect humans and animals has not yet been adequately addressed.

These uncertainties raise the following specific questions. Does the natural diversity and broad population structure of *S. enterica* differ between continents, or by source? Are *S. enterica* populations uniform across smaller geographic entities with multiple legal entities but continuous contact, such as the island of Ireland? Do isolates from water and reptiles cause gastroenteritis in humans? A broad sampling of *Salmonella* from diverse geographical sources and multiple hosts is needed to answer these questions, and to counteract the current extreme bias in the public databases of *Salmonella* genomes.

Between 2007 and 2012, the authors of this manuscript and their colleagues (see Acknowledgements) shared representative isolates of *S. enterica* from their strain collections with MA at University College Cork in order to address these questions. Single colony isolates were cultivated and stored frozen in robotic instrumentation-friendly vials in microwell-format storage racks. At that time, the primary sequence-based genotyping for large collections was classical MultiLocus Sequence Typing (7-gene MLST) ( Kidgell *et al.*, 2002; Maiden *et al.*, 1998) ( Box 1), and several thousand isolates from the strain collection were subjected to this procedure ( Achtman *et al.*, 2012; Zhou *et al.*, 2020a). These analyses did not extend to the entire strain collection, and it has therefore not been previously described in detail. The entire collection accompanied MA to University of Warwick in 2013, and is now being maintained for posterity as “the Achtman collection” by Jay Hinton, University of Liverpool.

**Box 1. Explanations of acronyms and specialized designations**

MLST: MultiLocus Sequence Typing in which each sequence variant of a gene is assigned a unique numerical designation. The Sequence Type (ST) is the set of the allelic numbers for an individual strain or genome, and is also assigned a unique ST number. e.g. ST4 might consist of alleles 1 2 1 1 3 5 1. First described for *Neisseria meningitidis* in 1998 and now extended to a large number of bacterial species ( Jolley *et al.*, 2018).

7-gene MLST ( *S. enterica*): Classical MLST involving 7 housekeeping genes ( Achtman *et al.*, 2012; Kidgell *et al.*, 2002). STs are grouped together in eBurst Groups (eBGs) based on minimal spanning trees, which correspond to serovars and are curated manually.

wgMLST ( *Salmonella*): Whole genome MLST based on 21,065 genes from a pan-genome based on 537 representative *Salmonella* genomes ( Alikhan *et al.*, 2018).

cgMLST ( *Salmonella*): Core-genome MLST based on a subset of 3002 genes from the wgMLST scheme that were present in ≥98%, intact in ≥94% and of unexceptional diversity in 3144 representative *Salmonella* genomes ( Alikhan *et al.*, 2018). STs are referred to as cgSTs.

Lineage: A deep branch in a phylogenetic tree which seems to represents a distinct monophyletic group according to visual examination.

HierCC: Single linkage hierarchical clustering of cgSTs based on a maximal internal distance of a certain number of different alleles in pairwise comparisons ( Zhou *et al.*, 2020b). HC100, HC900, HC2000: hierarchical clusters with maximal length of internal branches of 100, 900 and 2000 alleles. HC900 is roughly equivalent to eBGs, but more reliable due to the higher resolution. HC2000 roughly equates to Lineages, except that HC2000 is based on a network approach with a defined algorithm whereas Lineage designations are based on trees and are subjective.

Genomic sequencing of large numbers of samples has recently become feasible even for modestly-sized research groups ( Loman *et al.*, 2012), as documented by the recent sequencing of several thousand genomes from extra-intestinal human infections with non-typhoidal *Salmonella* in the Americas and Africa ( Perez-Sepulveda *et al.*, 2020). Here we provide an overview of the UoWUCC (University of Warwick/University College Cork) 10K genomes project, in which 9769 *S. enterica* genomes were sequenced from strains in the Achtman collection in order to address the questions posed above.

## Results

**Figure 1.**
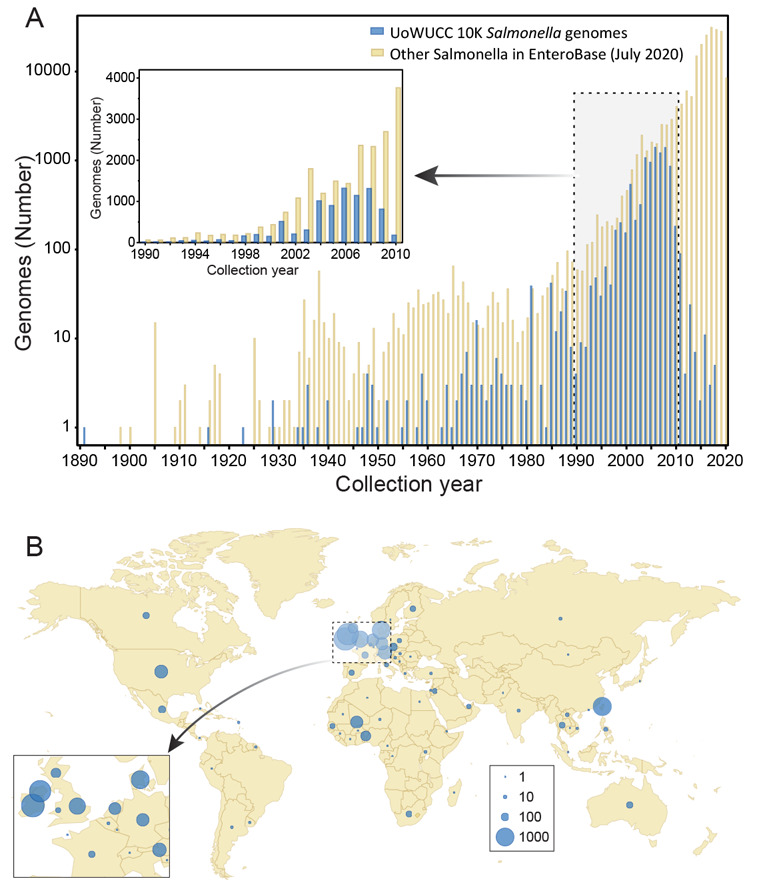
Sources of bacterial isolates for the 10K UoWUCC *Salmonella* Genomes Project. **A**) Semi-logarithmic histogram of numbers of genomes in EnteroBase by year of isolation. Genomes from the 10K project with known dates of isolation are shown in blue and other *Salmonella* genomes in yellow. Inset: Genomes which were isolated between 1990 and 2010. **B**) Geographic distribution of sources of isolation. Dot circles are proportional to numbers of strains as indicated in the Key legend at the lower right. Inset: Expanded map of the region near the English Channel.

**Themes within the 10K genomes project.** Table 1 provides an overview of the sources of most of the bacterial isolates whose genomes were sequenced, grouped into sub-collections according to theme. The “Rivers” theme includes 466 isolates from rivers in the United States and England, as well as from drinking water and faecal samples from healthy individuals in central Benin, Africa. The “Ireland” collection of 3880 strains were isolated from humans, livestock and food: 2125 from the Republic of Ireland and 1755 from Northern Ireland. We also sequenced 1131 isolates from Taiwan which represented the PFGE diversity of multiple *Salmonella* serovars from humans and reptiles. The “Reptiles” theme consisted of 794 other isolates from Austria, Australia, the Netherlands, Germany and Finland from serovars that infect both reptiles and humans. Finally, 3320 isolates were sequenced to cover “General diversity”, including non-Typhi isolates from long-term human carriers in Germany; reference strains for phage types of serovars Enteritidis and Typhimurium; diverse veterinary isolates from England; and Typhimurium from the mesenteric lymph nodes of asymptomatic pigs in Canada. The “General diversity” sub-collection also included members of the SARA and SARB collections as well as human isolates from diverse global sources. The UoWUCC 10K collection spans the time frame from 1891 to 2018 ( Figure 1A), but 94% (9206/9769) of its strains were isolated before 2011. It also spans a wide range of geographic diversity, and the bacteria were isolated from 73 countries on all the continents except Antarctica ( Figure 1B).

**Table 1.** Sources of 9591 *Salmonella* isolates that were sequenced within the UoWUCC 10K genomes project.

Themes and Sources	Number	Description
**Rivers**	Total: **466**	
A. Boehm (Stanford)	19	Central California rivers ( Walters *et al.*, 2011; Walters *et al.*, 2013)
R. Meinersmann (USDA)	188	Upper Oconee river, Georgia ( Meinersmann *et al.*, 2008)
A. Uesbeck (Univ. of Cologne)	177	Drinking water in wells and ponds, Benin ( Uesbeck, 2009)
J. Wain (HPA, Colindale)	82	Thames River. England
**Republic of Ireland**	Total: **2125**	
A. Coffey (CIT)	61	Food
M. Murphy (Cork County Vet lab)	67	Livestock, County Cork
D. Bolton (Teagasc, AFRC)	37	Livestock, bovine
D. Prendergast (DAFM)	479	Domesticated animals and food
M. Cormican (NSRL, Galway)	1126	Human
N. Leonard (UCD, Dublin)	317	Porcine
S. Fanning (UCD, Dublin)	38	Environment
**Northern Ireland**	Total: **1755**	
J. Moore (Belfast City Hospital)	899	Human
S. Strain (AFBI, Belfast)	449	Animal Health
B. Madden (AFBI, Belfast)	407	Agri-Food
**Taiwan**	Total: **1131**	
Chao-Chin Chang (SVM, NCHU)	48	Reptile isolates
Chien-Shun Chiou (CDC)	1083	Human isolates
**Reptiles and human** **isolates of the same** **serovar**	Total: **794**	
C. Kornschober (Austria)	366	Austria
D. Gordon (Canberra)	15	Australian deserts ( Parsons *et al.*, 2011)
X. Huijsdens (RIVM)	296	Netherlands
R. Helmuth (BfR Berlin)	85	Germany ( Achtman *et al.*, 2012)
S. Pelkonen (EVIRA, Finland)	32	Finland
**General diversity**	Total: **3320**	
Roy Curtiss 3rd	33	Human carrier strains, Germany, 1980s
S. Porwollik (SKCC)	25	General diversity ( Porwollik *et al.*, 2004)
E. De Pinna (HPA)	90	Enteritidis Phage type references, UK ( Ward *et al.*, 1987)
H.L. Andrews- Polymenis	52	Typhimurium Phage type references, Germany ( Andrews-Polymenis *et al.*, 2004)
G. Wise (VLA, Weybridge)	436	*A*nimals, England
S. Quessy (McGill)	18	Mesenteric lymph nodes from asymptomatic swine, Canada ( Perron *et al.*, 2008)
F. Boyd	61	Original SARA/SARB ( Achtman *et al.*, 2013)
L. Harrison (Univ of Pittsburgh)	314	Humans, Global ( Krauland *et al.*, 2009)
J. Wain (HPA, Colindale)	103	Humans, England ( Achtman *et al.*, 2012)
F.-X. Weill (Institut Pasteur)	137	Humans, France ( Achtman *et al.*, 2012)
W. Rabsch (Robert Koch-Institut, Wernigerode)	232	Humans, Germany ( Achtman *et al.*, 2012)
Z. Jaradat (JUST, Jordan)	23	Humans, Jordan
M. Zaidi (Mexico)	64	Humans, Mexico ( Wiesner *et al.*, 2009)
R. Kingsley (Sanger)	389	Humans, Mali ( Tapia *et al.*, 2015)
J. Bouldin (USDA)	29	Virulent Enteritidis
C. Kornschober (Austria)	86	Boar & Swine, Choleraesuis, Austria
U. Methner (Friedrich- Loeffler-Institut)	28	Boar & Swine, Choleraesuis, Germany ( Methner *et al.*, 2010)
I. Rychlik (VRI, Czech Republic)	86	Human, Typhimurium, Czech Republic ( Matiasovicova *et al.*, 2007)
E. Litrup & M. Torpdahl (SSI, Denmark)	1036	Human, 1 strain per MLVA type of Typhimurium , Denmark ( Lindstedt *et al.*, 2007)
N. Williams (University of Liverpool - IIGH)	78	Badger, Agama

UowUCC: University of Warwick/University College Cork.

**Figure 2.**
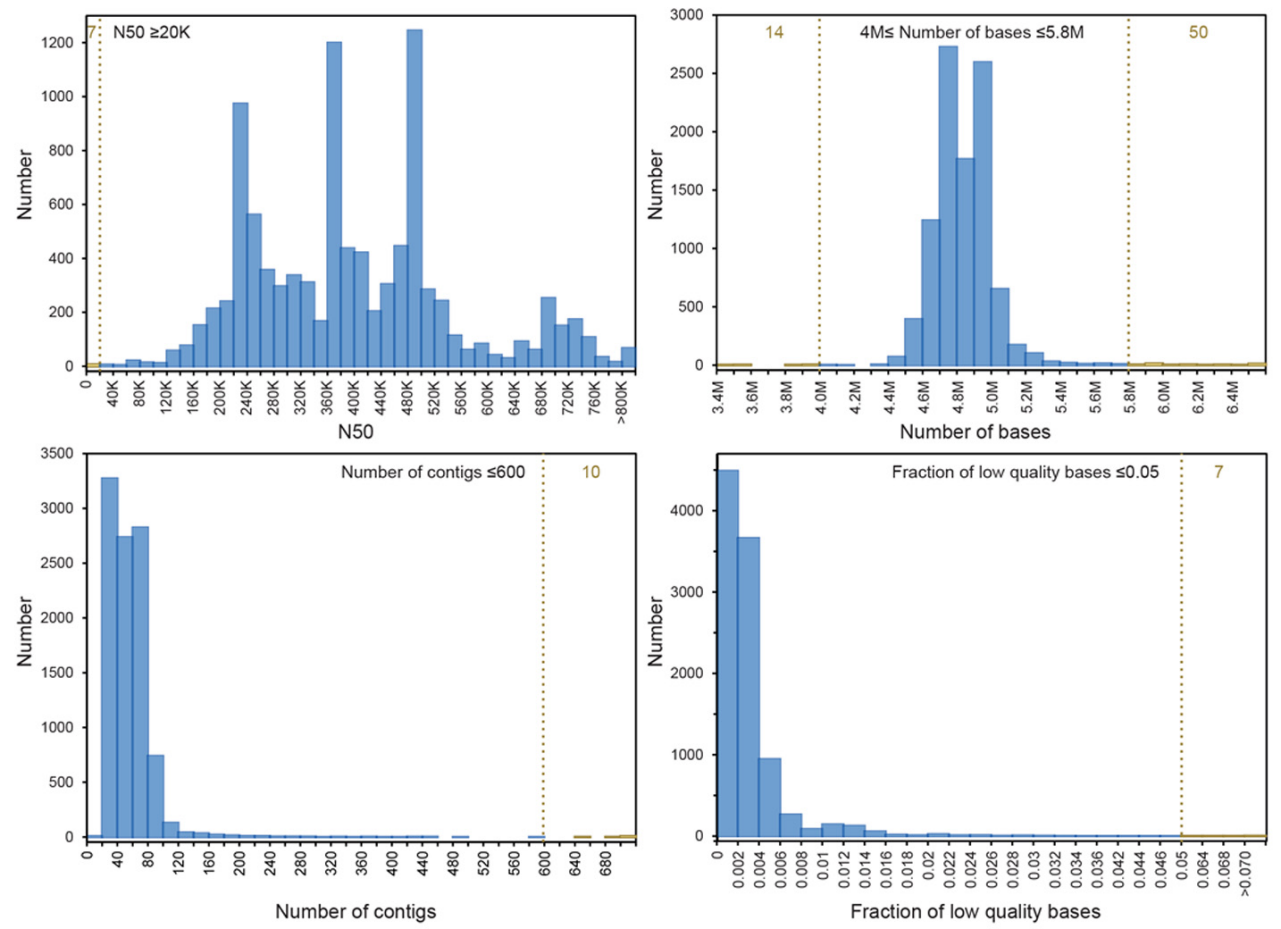
Quality control of 10K genomes. Default EnteroBase criteria are indicated by vertical dashed lines. Numbers of genomes in the 10K project which passed these cut-off criteria are indicated in blue and failures in yellow, with the total numbers of failures near the tops of the figures in yellow. The quality criteria consisted of N50 ≥20,000, genomic assembly size between 4 MB and 5.8 MB, a maximum of 600 contigs and a low fraction of uncalled, low quality bases (N’s).

**Sequence reads, genomes, genotypes and metadata.** After Illumina short read sequencing (see Methods), the sequence data files were uploaded to the Short Reads Archive at EBI, where they are publicly available for downloading. Genomes were assembled within EnteroBase using its standard pipelines ( Zhou *et al.*, 2020a), and the 9769 genome assemblies that passed stringent quality control criteria ( Figure 2) and manual curation ( Table 2) are publicly available via EnteroBase for inspection, analysis and downloading. EnteroBase also contains the relevant metadata, serovar predictions and MLST genotype assignments for classical 7-gene MLST (STs) ( Achtman *et al.*, 2012; Maiden *et al.*, 1998), ribosomal gene MLST ( Alikhan *et al.*, 2018; Jolley *et al.*, 2012), core genome MLST (cgMLST, cgSTs) ( Alikhan *et al.*, 2018; Zhou *et al.*, 2020a) and whole genome MLST ( Zhou *et al.*, 2020a) ( Box 1). The 10K genomes collection is identified by “M. Achtman” in the metadata field “Lab Contact”, and the original sources of the bacterial strains are listed in the metadata field “Comments”.

**Table 2.** Summary of the fate of 10,316 sets of short reads.

Category	Number of records
**Failed Quality** **Control**	**129**
**Mix-up/** **contamination**	**418**
Inconsistent MLST type	11
Inconsistent Serovar	374
Entire microwell plate(s)	33
**Final dataset**	**9769**
Consistent MLST ST	1801
Consistent serovar	7713
No independent verification	255

NOTE: The table ignores 1208 DNA samples which failed quality control at the Sanger Institute, and were not sequenced. New DNAs for 724 of them passed QC and are included in the table.

**Figure 3.**
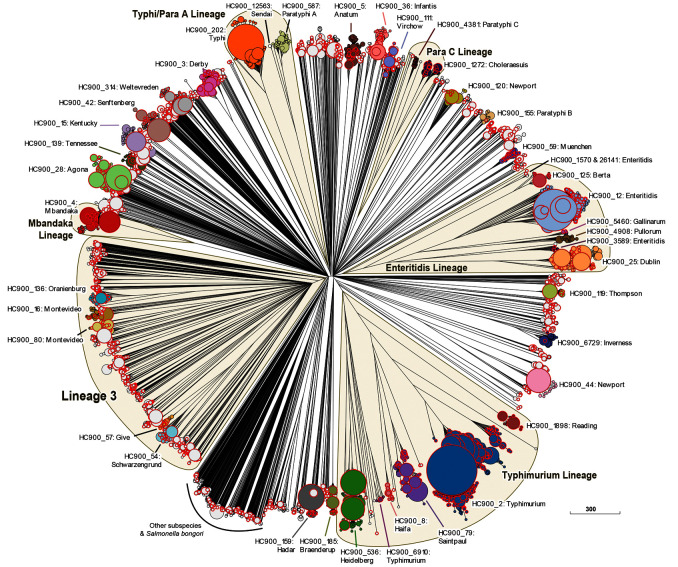
Genomic diversity of 33,052 pre-2011 genomes in EnteroBase, including 9206 from the 10K genome project (red perimeters). The figure shows a Ninja NJ ( Wheeler, 2009) tree of the numbers of different alleles between cgSTs as generated within EnteroBase using GrapeTree ( Zhou *et al.*, 2018a). Nodes from 41 common HC900 clusters are indicated by distinct colors, HC900 designations and predominant serovars. Lineages of HC900 clusters are indicated in yellow. The Enteritidis and Typhimurium Lineages are explored in greater detail in Figure 4 and the Mbandaka Lineage in Figure 5. Node sizes are proportional to the numbers of genomes they include. Nodes that include genomes from the 10K genomes project are highlighted by red perimeter. An interactive version can be found at http://enterobase.warwick.ac.uk/a/46053, in which the user can use other metadata for coloring genomes. Scale bar: 300 alleles.

**General overview of population structures.** The 10K collection accounts for 28% (9206/33,052) of all *Salmonella* genomes in EnteroBase (3 Aug 2020) whose strains had been isolated before 2011. Previously, 7-gene MLST STs were clustered in eBurst groups (eBGs) ( Box 1) which correlate strongly with serovar ( Achtman *et al.*, 2012; Alikhan *et al.*, 2018). STs are now being replaced by cgSTs (3002 genes) ( Box 1), which offer a broad range of resolution that is informative over the entire range from epidemiological tracking of micro-clades up to the sub-division of species at the genus level. eBGs are being replaced by hierarchical clusters of cgSTs (HierCC) in which internal branches can differ by up to 900 alleles (HC900 clusters) ( Zhou *et al.*, 2020b) ( Box 1). HC900 clusters provide higher resolution than eBGs, are more accurate and their cgST assignments remain stable even after the addition of large numbers of new genomes ( Alikhan *et al.*, 2018). Figure 3 shows the broad range of core genomic diversity which is present in the 33,052 pre-2011 genomes. These data demonstrate that the 10K genomes are broadly representative of all HC900 clusters in EnteroBase with only few exceptions. The exceptions include serovars Typhi, Paratyphi A and Paratyphi C which were not addressed because they had already been extensively investigated elsewhere ( Wong *et al.*, 2015; Wong *et al.*, 2016; Zhou *et al.*, 2014; Zhou *et al.*, 2018b), and several other serovars were not sequenced because they were rare in the sampled countries.

Similar to eBGs, most HC900 hierarchical clusters are associated with a single predominant serovar. Many HC900 clusters correspond to distinct clades, and share only very few alleles with any other HC900 cluster, resulting in an almost star-like phylogeny for many serovars ( Figure 3). However, some HC900 clusters do share some identical allelic sequences, allowing higher order phylogenetic relationships to be resolved for those lineages ( Box 1). One such Lineage is Lineage 3/Clade B ( Achtman *et al.*, 2012; den Bakker *et al.*, 2011; Didelot *et al.*, 2011; Parsons *et al.*, 2011) which encompasses multiple polyphyletic serovars that undergo inter-serovar recombination. Lineage 3 is clearly delineated in Figure 3, and the data confirm that it encompasses multiple HC900 clusters. The tree confirms other previously described, high level relationships such as the Typhi/Para A Lineage containing HC900 clusters corresponding to serovars Typhi, Paratyphi A and Sendai ( Didelot *et al.*, 2007), and the Para C Lineage containing HC900 clusters corresponding to serovars Paratyphi C, Choleraesuis, Typhisuis, Lomita and Birkenhead ( Key *et al.*, 2020; Zhou *et al.*, 2018b). However, Figure 3 aalso includes other poorly described, higher order lineages that each encompass multiple HC900 clusters and their serovars, including the Typhimurium and Enteritidis Lineages.

**Figure 4.**
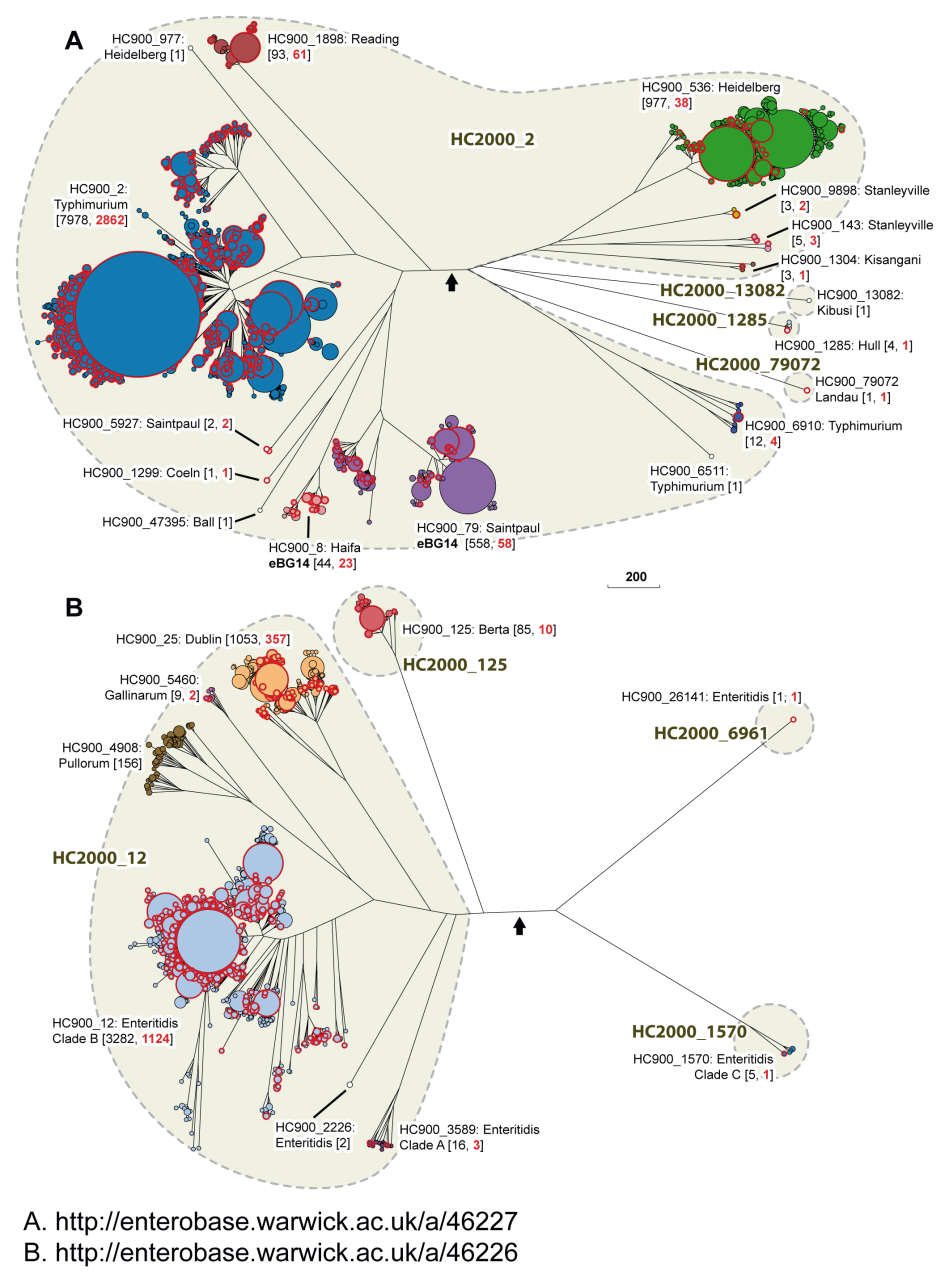
Detailed representations of HC 2000 and 900 clusters in the Typhimurium Lineage ( **A**) and the Enteritidis Lineage ( **B**). Each consists of a NINJA NJ tree of the subset of nodes encompassed by the corresponding Lineages from the tree in Figure 3. The figure indicates HC2000 clusters in larger font and gray shading. Designations for individual HC900 clusters and their predominant serovar include the total number of isolates (black) and the number from the 10K genomes project (red) in parentheses. In part B, Clade A and C designations from citations ( Graham *et al.*, 2018; Luo *et al.*, 2020) are indicated for HC900_3589 and HC2000_1570, respectively. Interactive versions can be found at http://enterobase.warwick.ac.uk/a/46227 ( **A**) and http://enterobase.warwick.ac.uk/a/46226 ( **B**), in which the user can use other metadata for coloring genomes. Black arrowheads: tree root. Scale bar: 200 alleles.

**Typhimurium Lineage.** In 1991, the SARA strain collection of 72 representatives of the so-called *“S. typhimurium complex”* was chosen on the basis of multilocus enzyme electrophoretic typing ( Beltran *et al.*, 1991). SARA includes representatives of serovars Typhimurium, Saintpaul, Heidelberg, Paratyphi B/Java and Muenchen. The Typhimurium Lineage defined by cgMLST also encompasses serovars Typhimurium, Saintpaul, and Heidelberg ( Figure 3), but not Paratyphi B/Java or Muenchen, which are quite distinct in Maximum Likelihood trees of core SNPs ( Zhou *et al.*, 2018b). The genomes in the Typhimurium Lineage define multiple HC2000 hierarchical clusters: HC2000_2, HC2000_13082, HC2000_1285 and HC2000_79072 ( Box 1) ( Figure 4A). Many of the serovars in the Typhimurium Lineage are polyphyletic, and fall into multiple HC900 clusters within HC2000_2 (Typhimurium: HC900_2, HC900_6511 and HC900_6910; Heidelberg: HC900_536, HC900_977; Saintpaul: HC900_79, HC900_5927; Stanleyville: HC900_143, HC900_9898), which are intermingled in the tree with still other HC900 clusters of serovars Reading, Coeln, Ball, Haifa, and Kisangani ( Figure 4A). The other HC2000 clusters include a few strains each from serovars Kibusi, Hull and Landau, and each consists of a single HC900 cluster. MLST clustering of *Salmonella* based on assignments to 7-gene STs ( Achtman *et al.*, 2012) has been widely used ( Bawn *et al.*, 2020; Cheng *et al.*, 2019). The resolution of such MLST typing is limited and the relationship of STs to HierCC clusters is not necessarily uniform. For example, almost all HC900_1898 (Reading) genomes belong to ST1628, and almost all HC900_536 (Heidelberg) genomes are ST15. However, HC900_79 (Saintpaul) contains multiple common STs (27, 50, 680 and others). And the main Typhimurium cluster, HC900_2, is predominantly ST19 but also contains ST34, ST36, and ST313, which correspond to distinct HC100 or HC400 internal clusters. We conclude that the results presented here provide an unprecedented overview of the high order population structure of the Typhimurium Lineage and note that additional analyses will be needed to elucidate the internal structure of individual HC900 clusters at higher resolution. Our preliminary analyses indicated that the evolutionary history of HC2000_2 is likely to have been complicated and involved multiple recombinational events. Elucidating this history will be facilitated by the genomes in the 10K genomes collection because they straddle the entire diversity just described.

**Enteritidis Lineage.** The Enteritidis Lineage ( Figure 3) includes one predominant HC2000 cluster, HC2000_12, as well as three smaller HC2000 clusters. HC2000_12 includes HC900_12, which contains most of the genomes of serovar Enteritidis strains from Europe, North America and Africa, as well as one HC900 cluster for each of the related serovars ( Feasey *et al.*, 2016; Langridge *et al.*, 2015) Gallinarum (HC900_5460), Pullorum (HC900_4908) and Dublin (HC900_25) ( Figure 4B). HC2000_12 also includes two other HC900 clusters of serovar Enteritidis (HC900_2226 and HC900_3589), which are more distinct from HC900_12, the major Enteritidis cluster, than are the Pullorum, Gallinarum or Dublin clusters. The Enteritidis Lineage contains a second HC2000 cluster for serovar Berta (HC2000_125), and two additional clusters of Enteritidis (HC2000_6961, HC2000_1570).

Recent analyses have separated Enteritidis into clade B, which corresponds to HC900_12, and two other distinct clades of Enteritidis, A and C, which are common in Australia ( Graham *et al.*, 2018; Luo *et al.*, 2020). (These were originally referred to as lineages but clades are substituted here to prevent confusion with the Lineages in Figure 3). Clade A corresponds to HC900_3589, which is part of HC2000_12, and clade C to HC2000_1570 ( Figure 4B). There are currently a total of five Enteritidis clades within the Enteritidis Lineage ( Figure 4B). Similar to the Typhimurium Lineage, Enteritidis and related serovars are polyphyletic and likely reflect a complicated evolutionary history.

The 10K genomes are distributed across the breadth of the entire Enteritidis lineage, except for Pullorum, which has largely been eradicated from the countries that were sampled ( Le Bouquin *et al.*, 2020). Interestingly, the 10K genomes collection also includes old isolates of Enteritidis clades A and C which are currently particularly common in Australia. Strain E2387 in HC2000_1570 (clade C) is the original reference strain for phage type PT14, and was isolated in England in 1968, long before any descriptions of clade C in Australia. The 10K collection also includes three older strains in HC900_3589 (clade A): strain P106993, the reference strain for PT26, was isolated in England in 1987, and the recent Australian clade A isolates were also PT26. Two other HC900_3589 strains were isolated from snakes in Germany in 2002 and 2003. Similarly, the sole genome in HC2000_6961 is the reference strain for PT11b, strain PT187803, which was isolated in Canada in 1989. 

Similar to Typhimurium, the primary Enteritidis cluster, HC900_12 largely consists of a single 7-gene ST, ST11. According to cgMLST and HierCC, most HC900_12 genomes are associated with HC100_87 and HC100_12, but it also contains at least eight additional, distinct tight clusters (HC100_12675, 2452, 13575, 447, 12703, 1522, 1404, 31939). Some of these correspond to 7-gene STs such as ST136 (HC200_12703) and ST183 (HC200_1404) ( Achtman *et al.*, 2012; Langridge *et al.*, 2015) or the geographically associated Enteritidis lineages within SNP trees designated as the Central/Eastern Africa (HC100_12675) and West African (HC100_2452) lineages ( Feasey *et al.*, 2016). The others do not seem to have been previously described. Once again, the availability of the UoWUCC genomes will assist future reconstructions of global diversification and dispersion of individual lineages.

**Figure 5.**
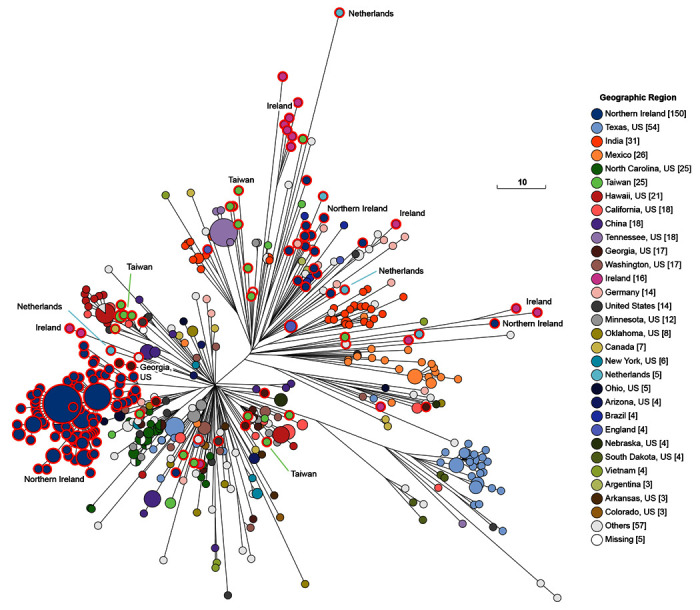
Genomic diversity of 601 pre-2011 genomes from HC100_4 of which 208 were from the 10K genomes project (red perimeters). The figure shows a Ninja NJ ( Wheeler, 2009) tree of the numbers of different alleles between cgSTs as generated within EnteroBase using GrapeTree ( Zhou *et al.*, 2018a). The geographical sources of some of the isolates from the 10K genomes project are indicated to demonstrate that multiple micro-clades were present in individual countries. An interactive version can be found at http://enterobase.warwick.ac.uk/a/46139, in which the user can use other metadata for coloring genomes. The same tree colored by general source can be found in Figure 6 and a tree showing all modern Mbandaka and Lubbock genomes can be found in Figure 7. Scale bar: 10 alleles. Color Key at right.

**Figure 6.**
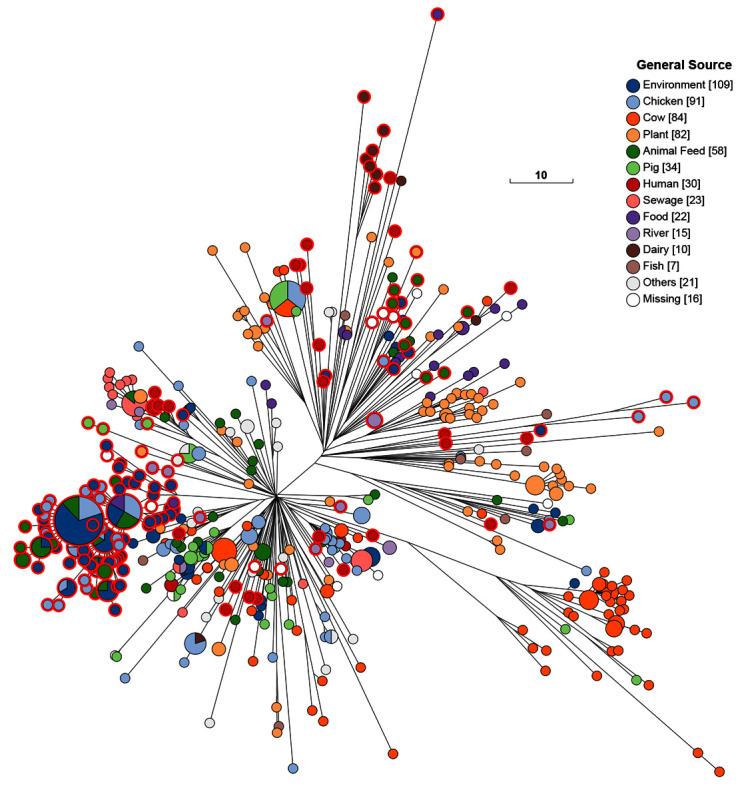
As Figure 5, except that the nodes are colored by general source.

**Mbandaka Lineage.** The 10K genomes are also likely to be useful for fine-scale analyses within clades with even more limited genetic diversity. We provide an initial example of this utility by zooming in on the Mbandaka Lineage ( Figure 3). Serovar Mbandaka was first isolated in 1948 but has now become a common source of salmonellosis in humans in the EU and elsewhere ( Cheng *et al.*, 2019; Hoszowski *et al.*, 2016). Examination of the sources of the genomes of the Mbandaka Lineage up to 2010 ( Figure 5) provides a different perspective because most were from environmental samples, animal feed, sewage, rivers and dairy products with a smaller proportion from chickens, cows, plants, pigs and humans ( Figure 6). Thus, Mbandaka seems to be commonly shed to the environment by livestock rather than being a primary human pathogen.

**Figure 7.**
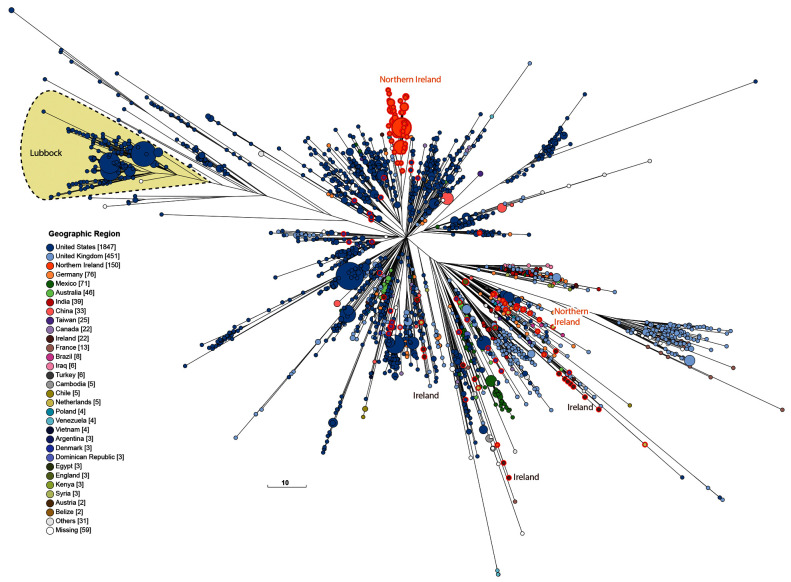
Genomic diversity of 2955 genomes from HC100_4 from EnteroBase (18/08/2020) of which 208 were from the 10K genomes project (red perimeters). The figure shows a Ninja NJ ( Wheeler, 2009) tree of the numbers of different alleles between cgSTs as generated within EnteroBase using GrapeTree ( Zhou *et al.*, 2018a). The geographical sources of all isolates are color-coded (Key at lower left) and the location of serovar Lubbock is shaded. Unshaded isolates are serovar Mbandaka. An interactive version can be found at http://enterobase.warwick.ac.uk/a/46122, in which the user can use other metadata for coloring genomes. Scale bar: 10 alleles.

The Mbandaka Lineage shows so little diversity that almost all of its genomes are included in the tight HC100_4 cluster ( Figure 5 and Figure 6), which has a maximal internal branch length of 100 different alleles. Mbandaka cgMLST genotypes cluster very tightly by geographic source and by host, yielding fairly uniform clusters of isolates from cows, plants, dairy products, and chicken farms (chickens plus environmental swabs) ( Figure 6). In 2015, a recombinational variant of Mbandaka was designated as serovar Lubbock ( Bugarel *et al.*, 2015). Figure 7 shows the current composition of HC100_4, in which Lubbock constitutes a micro-clade. Even today, almost all clades are country-specific, but each country contains multiple micro-clades.

The 10K genomes project provided 25% (208/601) of the H100_4 genomes in EnteroBase that were isolated prior to 2011. These 208 genomes were from multiple themes in Table 1, from diverse geographical sources, and were scattered throughout the cgST tree among isolates from other global sources ( Figure 5). Most of the 16 Mbandaka bacterial strains from the Republic of Ireland were from dairy products, humans and pigs. Northern Ireland was the source of 151 other Mbandaka strains, predominantly from chicken farms and animal feed. Multiple micro-clades from each of these two geographic sources were inter-dispersed among other Mbandaka genomes. However, the genomes from Ireland did not cluster together with those from Northern Ireland even though their geographic sources are at most a few hundred kilometers apart. Exceptionally, one genome from Ireland (a chicken isolate) clustered tightly with genomes from Northern Ireland. The primary clades found in Ireland and Northern Ireland were not found in any other country, and they have remained genetically discrete from other geographical sources up to recent times (18 Aug 2020) when HC100_4 contained 2955 genomes of serovars Mbandaka and Lubbock ( Figure 7). Most of the additional strains isolated since 2011 are from the US or the UK, and show broad continental specificity, interspersed with the isolates from the 10K collection which are spread throughout the entire Mbandaka tree.

## Discussion

**One Health approach**. MA initiated a broadly-based collection of *Salmonella* from diverse sources in 2008. At the same time, the One Health Initiative ( Kahn *et al.*, 2020) independently proposed combining global epidemiological and other information about pathogens that originated from human and animal infections, as well as from the environment. For the last few years, comparisons of bacterial isolates from multiple sources have been pursued for *Salmonella* and other food-borne pathogens by the Food and Drug Administration in the United States, which has sequenced numerous bacterial strains isolated from plants and the environment in addition to food samples. The FDA has also been exemplary in sequencing genomes from around the globe, and in establishing the GenomeTrakr website to provide access to those genomes and their properties ( Timme *et al.*, 2019). GenomeTrakr also includes numerous genomes of human isolates that have been sequenced by the Sanger Institute and the CDC. Unfortunately, most of these entries lack the metadata required to inferface with information from food and environmental sources. The genome sequencing efforts by Public Health England since 2015 are also highly laudable, and they publish short reads together with the corresponding metadata from all human *Salmonella* isolates from England and Wales in the ENA Sequence Reads Archives ( Ashton *et al.*, 2016; Waldram *et al.*, 2018). However, very few genomes of *Salmonella* from non-human sources in England are publicly available, and the rest of Europe is only now beginning to sequence and publish genomic sequence reads and their metadata. Furthermore, most European countries continue to maintain separate networks of laboratories for isolates from humans and from domesticated animals or food, with the two networks being separately coordinated by ECDC and EFSA. These two organisations have not yet implemented universal genomic sequencing (EFSA (European Food Safety Authority) and ECDC (European Centre for Disease Prevention and Control), 2019; ECDC (European Centre for Disease Prevention and Control) *et al.*, 2019), and are not yet actively supporting it. Thus, the aims of the One Health Initiative are not being adequately met for *Salmonella,* and the completion of the UoWUCC 10K *Salmonella* genomes project is a major step forward towards those goals.

**Accuracy.** According to our experience, a few percent of isolates from all reference/diagnostic laboratories are incorrectly serotyped ( Achtman *et al.*, 2012). Sporadic curation of EnteroBase has also revealed numerous instances where the metadata in the short read archives were inconsistent with the serovars that were predicted from the assembled sequences. Such discrepancies likely reflect laboratory mistakes or typographical errors and/or data transmission glitches. We manually curate such discrepancies in EnteroBase when we notice them. In several cases we have deleted the genomes. However, we usually simply replace obviously false serovars with the predicted serovars from the genomic assemblies ( Robertson *et al.*, 2018; Zhang *et al.*, 2019), and currently almost 20% of the serovar metadata for *Salmonella* in EnteroBase are based on such predictions. For other cases we have replaced false metadata with the corresponding published data, e.g. for the Murray collection ( Baker *et al.*, 2015).

The SARA ( Beltran *et al.*, 1991) and SARB ( Boyd *et al.*, 1993) collections are invaluable reference sets for the genetic diversity of the serovars that they represent, but these collections are badly contaminated in multiple laboratories ( Achtman *et al.*, 2013), and many of their supposed genomes in the public domain were sequenced from contaminants. We sequenced a clean set of those strains ( Achtman *et al.*, 2013), and ensured that public genomes from contaminated variants were either deleted from EnteroBase, or were relegated to the category of sub-strains ( Zhou *et al.*, 2020a), which are not visible without special intent. However, there are too many sets of short reads in the public domain to manually correct all of them, and EnteroBase perpetuates numerous false metadata that accompanied short reads.

The metadata for the 10K genomes are much more accurate than is the rule for public genomes because we manually curated them for plausibility (see Methods), and only those that survived curation remain in EnteroBase ( Table 2). As a result, the 10K genomes are likely to contain fewer mistaken combinations of genomes and metadata than has been the norm.

**Historical reconstructions.** Possibly scientists that focus on contemporary outbreaks of human salmonellosis might argue that the 10K genomes are irrelevant because almost all those strains were isolated before 2011, and many even date back to the 1980s and earlier. Instead, many previous analyses of population patterns have been biased to isolates from a single country and/or a narrow range of years of isolation. A broad resource of older genomes will provide the historical background needed to reconstruct evolutionary patterns over decades and possibly even over centuries. For example, it was only possible to describe the evolutionary history over millennia of a *Salmonella* branch that includes serovar Paratyphi C ( Key *et al.*, 2020) because rare serovars had been sequenced within the 10K genomes project.

Several other dramatic examples of the value of historical isolates are provided here, e.g. old reference strains for phage types of Enteritidis from Europe that predated by decades the dates that related bacteria were isolated in Australia. Many public health laboratories are forced to discard older strains due to space constrictions, e.g. the clinical strains from the Republic of Ireland are no longer available except within the Achtman collection.

**Geographical diversity.** The strains analysed here are not only old; they also represent unique diversity that is not otherwise represented among the >275,000 *Salmonella* genomes currently in EnteroBase. One example are genomes of Agama from badgers in Woodchester Park in England, which are uniquely represented by genomes within this project and allowed the reconstruction of transmission chains between neighboring setts ( Zhou *et al.*, 2020a). Another important example is Mbandaka from chickens and chicken farms in Northern Ireland in the early 2000s. The only Mbandaka genomes in EnteroBase that stem from Northern Ireland are the 152 genomes in the 10K project, and in 2020 they still differed from all 2800 other Mbandaka/Lubbock genomes in EnteroBase

**Does natural diversity of *S. enterica* differ between continents, or by source?** *S. enterica* is a transmissible pathogen with multiple hosts. We therefore expected the 10K Genomes project to provide multiple additional examples of global transmissions and spread from diverse zoonotic and environmental sources to humans. One such example was finding old isolates of Enteritidis clades in Europe that were thought to be specific to Australia. Unexpectedly, we also found support for geographic and host-specificity, for example a specific clade of Mbandaka isolates among chickens in Ireland.

EnteroBase contains >275,000 *Salmonella* genomes, but most of them are from common serovars infecting humans in the US and the UK. The 10K genomes project has added numerous additional details to the global genetic and genomic diversity of *Salmonella.* In turn, that additional diversity warrants an extensive investigation of the entire dataset. However, such an ambitious project would exceed the capabilities of a small group of scientists, including the authors of this report on their own. We therefore heartily invite the entire global *Salmonella* community to join in this investigation.

## Methods

**Bacterial isolates**. *S. enterica* isolates from multiple sources were collected at University College Cork by MA from 2008–2012, and their metadata were stored in a BioNumerics (Biomerieux) database. The metadata included country, year, and source of isolation, but none of the details that might allow identification of individual farms or people from whom they were isolated. No ethical permissions are required for transfer of such bacterial samples.

Microbiological cultivation was performed as described in detail elsewhere ( O'Farrell *et al.*, 2012). Isolated single bacterial colonies were used to inoculate 1.4 ml growth/freezing medium in 2-D bar-coded, screw-capped FluidX tubes ( O'Farrell *et al.*, 2012) whose physical locations were stored in an ItemTracker database. These tubes were grown overnight with shaking at 37°C, and stored at -80°C. All subsequent operations were performed with automated microbiology as described ( O'Farrell *et al.*, 2012). Cross-contamination from other tubes with these automated methods is not detectable in the sub-cultures, but can occur at a frequency of 1/500 in the parental tubes. Therefore, whenever the stock tubes were used for DNA isolation of a particular isolate, the most recently frozen serial sub-culture was used to inoculate one new subculture for freezing and storage as well as a second subculture for DNA isolation. DNA was isolated from many of these strains, and subjected to classical 7-gene multilocus sequence typing (MLST) ( Achtman *et al.*, 2012; Achtman *et al.*, 2013; O'Farrell *et al.*, 2012).

The strain collection, robotic equipment and databases accompanied MA to the University of Warwick in 2013, where the same procedures were implemented, except that DNA isolation was performed with a Qiagen QiaCube. We chose over 10,000 isolates of *S. enterica* for genome sequencing ( Table 1 and Table 2), with priority given to isolates whose DNA had previously been isolated and 7-gene MLST performed. Once those samples had been processed, DNAs were isolated from additional strains in the collections in Table 1. DNA concentrations were calibrated with Pico Green fluorescence to ensure that each sample contained at least 400 ng of DNA. Each sample was diluted into two 0.5 ml FluidX screw-capped, 2-D bar-coded tubes. One set of duplicate tubes was shipped to the Sanger Institute, Hinxton, UK for draft genome sequencing, and the second was maintained as a reserve at University of Warwick.

**Draft genome sequencing.** At the Sanger Institute, DNA samples were quantified once again, with a Biotium Accuclear Ultra high sensitivity dsDNA Quantitative kit using a Mosquito LV liquid handler, an Agilent Bravo WS automation system and a BMG FLUOstar Omega plate reader. DNAs which passed quality control were cherry-picked and diluted to 200 ng in 120 µl using a Tecan liquid handling platform. The microwell plates containing cherry-picked DNAs were sheared to 450 bp using a Covaris LE220 instrument.

Sheared samples were purified on the Agilent Bravo WS using Agencourt AMPure XP SPRI beads on a Beckman BioMek NX96 liquid handling platform. Library construction (end-repair, adapter-tailing and ligation) were then performed with an NEB Ultra II custom kit (Agilent Bravo WS), followed by PCR reactions to generate sequencing libraries using Kapa HiFi Hot start mix (Kapa Biosystems) and IDT 96 iPCR tag barcodes (IDT). The PCR cycles were: 95°C for 5 minutes; 6 cycles of 98°C for 30 seconds, 65°C for 30 seconds and 72°C for 2 minutes and were terminated by incubation at 72°C for 5 minutes. The IDT 96 iPCR barcodes consisted of the first 96 primers in the 384 set in Supplementary table S1 of Quail *et al*. ( Quail *et al.*, 2014). The resulting DNA was then purified again using Agencourt AMPure XP SPRI beads and quantified with the Biotium Accuclear Ultra high sensitivity dsDNA Quantitative kit. Libraries were pooled in equimolar amounts, 384 at a time, using a Beckman BioMek NX-8 liquid handling platform. The pooled libraries were normalised to 2.8 nM prior to cluster generation on an Illumina cBOT, and were then sequenced with paired ends (2 × 150 bp) on one lane of an Illumina HiSeq X 10.

**Post-sequencing procedures.** Sets of short reads were extracted from the storage system at the Sanger Institute with the “path-find” module ( Bio-Path-Find), and uploaded into EnteroBase together with the corresponding metadata that had been stored in the BioNumerics database. The short reads were assembled by EnteroBase using the then current backend pipelines (versions 3.61 - 4.1) ( Zhou *et al.*, 2020a). For those strains where 7-gene MLST had been performed, we also created an identical sub-strain except that the experimental field in EnteroBase for 7-gene MLST data was filled from the data in the BioNumerics database.

**Manual curation.** Manual curation of the assembled genomes was performed within EnteroBase to generate the most accurate dataset that was possible. Where the data were available, we compared the genome-derived predictions for each isolate with serotype assignments from laboratory experiments and/or historical MLST data. To this end, we created a custom view and user-defined fields that contained an arbitrary sequential Plate number for each rack of 96 tubes (95 DNAs plus a blank in microwell format, i.e. from A1 to H12) and information on the rows and columns of the tubes as well as their barcodes. We created one workspace for all the strains and their sub-strains for each microwell rack. 7-gene MLST data from the older ABI-based sequence data were compared with 7-gene MLST predictions from the genome assemblies. In initial comparisons, discrepancies between the two sources of data were pursued by inspecting the original sequence traces. However, all discrepancies reflected false calls of the ABI data. Thereafter, we treated discrepancies of up to one allele as indicating consistency, and discarded genomes with discrepancies of 2-7 alleles. For genomes without prior 7-gene MLST data, we compared the serovar based on agglutination tests with the serovars predicted from the genomic assemblies by SeqSero2 ( Zhang *et al.*, 2019), SISTR1 ( Robertson *et al.*, 2018) and 7-gene MLST eBurstGroups (eBGs) ( Achtman *et al.*, 2012). Discrepancies were examined for plausibility according to antigenic formulas ( Grimont & Weill, 2007), and genomes with gross discrepancies were discarded. Some 255 genomes lacked metadata on serovar but the remaining metadata on source and year of isolation was considered reliable, and these were kept despite the lack of independent confirmation of a lack of contamination. The numbers in these different categories are summarized in Table 2.

After excluding 129 assembled genomes that failed EnteroBase quality control criteria and 418 genomes with dramatically discrepant 7-gene MLST sequence types and/or serovar ( Table 2), we retained genomes from 9769 strains from the 10K collection ( http://enterobase.warwick.ac.uk/a/45743). The short sequence reads of the final set of strains were deposited in EBI.

**Analysis.** All analyses were performed within EnteroBase with the tools that were described by Zhou *et al.*, ( Zhou *et al.*, 2020a), as specified in the figure legends. All trees were created with the version of GrapeTree ( Zhou *et al.*, 2018a) that is integrated into EnteroBase, and can be interactively interrogated within EnteroBase. 

## Data availability

### Underlying data

Short read sequences are available for public access at the Short Reads Archive (SRA) at EBI under BioProject accessions PRJEB20997 and PRJEB33949.

NCBI BioProject: *Salmonella enterica* ancient DNA and modern demography. Accession number: PRJEB20997

NCBI BioProject: EnteroBase - User Uploads from M. Achtman to EnteroBase Salmonella database. Accession number: PRJEB33949

All other data is available for public access in EnteroBase http://enterobase.warwick.ac.uk in the *Salmonella* database. Individual strains and genomes from this project can be located with the same BioProject accession codes and also by the metadata field containing the text “M. Achtman”. All the analyses described were performed with software that are available within EnteroBase. Access to the individual trees with the option of colour-coding by other metadata is publicly accessible at: 

Figure 3: http://enterobase.warwick.ac.uk/ms_tree/46053;

Figure 4A: http://enterobase.warwick.ac.uk/ms_tree/46227;

Figure 4B: http://enterobase.warwick.ac.uk/ms_tree/46226;

Figure 5, 6: http://enterobase.warwick.ac.uk/ms_tree/46139;

Figure 7: http://enterobase.warwick.ac.uk/ms_tree/46122.
