# Supplementary material for: Genomic diversity of Salmonella enterica -The UoWUCC 10K genomes project
Source: Wellcome Open Res. 2021 Feb 1;5:223. Originally published 2020 Sep 24. [Version 2] doi: 10.12688/wellcomeopenres.16291.2 (PMC7869069; doi:10.12688/wellcomeopenres.16291.2)
Supplement: Peer review history

# Peer review: Reviewer response for version 2

**DOI:** 10.21956/wellcomeopenres.18280.r42391

**Published:** 2021-02-01

**Authors:** Jay C. D. Hinton

**Affiliations:** 1 Institute of Infection, Veterinary and Ecological Sciences, University of Liverpool, Liverpool, UK

I am happy with the revised version of the manuscript.

Is the work clearly and accurately presented and does it cite the current literature?

Yes

If applicable, is the statistical analysis and its interpretation appropriate?

Yes

Are all the source data underlying the results available to ensure full reproducibility?

Yes

Is the study design appropriate and is the work technically sound?

Yes

Are the conclusions drawn adequately supported by the results?

Yes

Are sufficient details of methods and analysis provided to allow replication by others?

Yes

Reviewer Expertise:

Salmonella functional genomics and gene regulation.

I confirm that I have read this submission and believe that I have an appropriate level of expertise to confirm that it is of an acceptable scientific standard.

# Peer review: Reviewer response for version 1

**DOI:** 10.21956/wellcomeopenres.17900.r40967

**Published:** 2020-12-18

**Authors:** Jay C. D. Hinton

**Affiliations:** 1 Institute of Infection, Veterinary and Ecological Sciences, University of Liverpool, Liverpool, UK

300,000 *Salmonella *genomes are now publicly available, meaning that more representatives of the *Salmonella* genus have been genome-sequenced than for any other bacterium. However, the majority of these genome-sequenced *Salmonella* isolates originated from humans or domesticated animals.

 In this major study, the Achtman group have assembled a fascinating collection of 10,000 *Salmonella* isolates that spanned more than a century, and were obtained from a wide range of mammalian, reptilian and environmental sources.

 By generating high-quality genome sequence, and doing analysis with the impressive EnteroBase resource, the paper not only provides invaluable genome-based information concerning the true diversity of the *Salmonella* genus, but also contributes new insights into the relatedness of the important Enteritidis and Typhimurium serovars. The focus on the Mbandaka Lineage is both timely and interesting.

 The manuscript is extremely well-written, and requires very few modifications. Some minor comments are listed below.

Minor comments:

- In the Introduction, I suggest that rather than citing the Kingsleyet al (2009) paper, a more recent publication is referred to Stanaway, JD *et al. *(2019) ^1^ *.*
- In the first paragraph of the “general overview of population structures” section, I wasn’t clear what the phrase “excluded from the 10 genomes” meant. Could this be clarified?
- In the second paragraph of the “general overview of population structures” section, please add an additional reference for Clade B such as den Bakker HC *et al.* (2011) ^2^.
- The scale bar described in legend to Figure 4 shows “200 alleles”. Is this the same as “200 SNPs”? If so, please use the term “SNPs” rather than “alleles” in this legend (and in other relevant figure Legends in the paper).
- At the beginning of the discussion, the term “catholic” is used to describe the UoWUCC collection. As the word “catholic” is not used as commonly as he used to be, I suggest it is changed to “wide-ranging” or similar.
- On page 10, change “all human *Salmonella* isolates in England” to “all human *Salmonella* isolates in England and Wales”.
- In the sentence that begins “In contrast to” on page 12, I was not clear what the words “accurate” and “plausibility” meant. Please rephrase.
- In the sentence that begins “As a result,” on page 12, I was not clear what the word “cleanest” meant. Please rephrase.
- On page 14, the important manual curation process is described. I suggest that an additional sentence is added at the beginning of this section to clarify the rationale of this approach for readers. One option would be to begin “Manual curation of the assembled genomes was performed within EnteroBase to generate the most accurate dataset possible. For individual genomes to be assigned to the final dataset, the genome-derived predictions needed to be consistent with either serotype or MLST data for each isolate. To this end…”, but of course the authors should make the sentence their own.

Is the work clearly and accurately presented and does it cite the current literature?

Yes

If applicable, is the statistical analysis and its interpretation appropriate?

Yes

Are all the source data underlying the results available to ensure full reproducibility?

Yes

Is the study design appropriate and is the work technically sound?

Yes

Are the conclusions drawn adequately supported by the results?

Yes

Are sufficient details of methods and analysis provided to allow replication by others?

Yes

Reviewer Expertise:

Salmonella functional genomics and gene regulation.

I confirm that I have read this submission and believe that I have an appropriate level of expertise to confirm that it is of an acceptable scientific standard.

## References

[1] : The global burden of non-typhoidal salmonella invasive disease: a systematic analysis for the Global Burden of Disease Study 2017. *The Lancet Infectious Diseases*.2019;19(12) : 10.1016/S1473-3099(19)30418-9 1312-1324 10.1016/S1473-3099(19)30418-9 31562022PMC6892270

[2] : Genome sequencing reveals diversification of virulence factor content and possible host adaptation in distinct subpopulations of Salmonella enterica. *BMC Genomics*.2011;12: 10.1186/1471-2164-12-425 425 10.1186/1471-2164-12-425 21859443PMC3176500

# Peer review: Reviewer response for version 1

**DOI:** 10.21956/wellcomeopenres.17900.r40964

**Published:** 2020-11-02

**Authors:** Xiangyu Deng

**Affiliations:** 1 Center for Food Safety, Department of Food Science and Technology, University of Georgia, Griffin, GA, USA

The authors reported a timely and laudable effort to substantially enrich publicly available genome data of Salmonella. This contribution is particularly valuable by 1) ameliorating the inherent and entrenched sampling bias toward certain countries and origins in public depositories of Salmonella genomes, and 2) accompanying genome resources with a powerful set of analytical and graphical tools as part of EnteroBase. 

 Taking the "Enteritidis Lineage" for example, existing epidemiology of Enteritidis is largely based on commonly circulating strains in North America and Europe, which often describes the population structure of the serotype as homogenous and clonal (although with the recognition of rare strains that are distantly related to the major Enteritidis clades). The UoWUCC 10K genomes project highlights the phylogenetic diversity of the serotype, as nicely demonstrated in the paper by an interactive figure that is easily accessible and highly customizable. 

 In the discussion, it would be helpful if the authors could explicitly answer or echo the four questions raised in the introduction (Does the natural diversity and broad population structure of *S. enterica* differ between continents, or by source? Are *S. enterica* populations uniform across smaller geographic entities with multiple legal entities but continuous contact, such as the island of Ireland? Do isolates from water and reptiles cause gastroenteritis in humans?)

 As a minor issue, certain source categories in Table 1 appear to overlap with each other, such as "livestock" and "domesticated animals". Some categories may need more precise definition, such as "environment".

Is the work clearly and accurately presented and does it cite the current literature?

Yes

If applicable, is the statistical analysis and its interpretation appropriate?

Yes

Are all the source data underlying the results available to ensure full reproducibility?

Yes

Is the study design appropriate and is the work technically sound?

Yes

Are the conclusions drawn adequately supported by the results?

Yes

Are sufficient details of methods and analysis provided to allow replication by others?

Yes

Reviewer Expertise:

Genomic epidemiology, Salmonella phylogenetics and evolution, food safety,

I confirm that I have read this submission and believe that I have an appropriate level of expertise to confirm that it is of an acceptable scientific standard.
